# Foot posture in female patients 5 years after breast-conserving surgery: a case–control study

**DOI:** 10.1007/s12282-018-0835-y

**Published:** 2018-01-24

**Authors:** Iwona Głowacka-Mrotek, Magdalena Sowa, Tomasz Nowikiewicz, Zygmunt Siedlecki, Wojciech Hagner, Wojciech Zegarski

**Affiliations:** 1 Department of Rehabilitation, Collegium Medicum of the Nicolaus Copernicus University in Torun, Bydgoszcz, Poland; 2 Department of Surgical Oncology, Collegium Medicum of the Nicolaus Copernicus University in Torun, Bydgoszcz, Poland; 3 Department of Laser Therapy and Physiotherapy, Collegium Medicum of the Nicolaus Copernicus University in Torun, Bydgoszcz, Poland; 4 Department of Clinical Breast Cancer and Reconstructive Surgery, Oncology Centre-Prof. F. Łukaszczyk Memorial Hospital, Bydgoszcz, Poland; 5 Department of Neurosurgery, Collegium Medicum of the Nicolaus Copernicus University in Torun, Bydgoszcz, Poland

**Keywords:** Feet, Breast-conserving therapy, Photogrammetric assessment, Adverse effects

## Abstract

**Purpose:**

Along with the improvement in the outcomes of breast cancer treatment being observed in the recent years, long-term studies to assess distant adverse effects of the treatment have become increasingly important. The objective of this study was to assess the foot posture in patients subjected to breast-conserving therapy. The assessment was made 5 years after the surgical procedure.

**Methods:**

116 female patients (mean age of 58.75 years) were qualified into a case–control study. Foot posture on the operated breast side (F1) as well as on the contralateral side (F2) was evaluated using a computer-based foot analysis tool as an extension of projection moiré-based podoscopic examination. Comparisons were made for the following parameters: limb load, L—foot length, W—foot width, L/W—Wejsflog index, ALPHA—hallux valgus angle, BETA—little toe varus angle, GAMMA—heel angle, KY—Sztriter–Godunov index, CL—Clarke’s angle, HW—heel width.

**Results:**

Five years after BCT, patients placed higher load on the foot on the side of the healthy breast (*p* = 0.0011). No statistically significant differences were observed between F1 and F2 with respect to other foot posture parameters (*p* > 0.05). No statistically significant differences were observed in foot posture parameters in patients having undergone BCT + ALND (axillary lymph node dissection) procedure as compared to patients subjected to BCT + SLNB (sentinel lymph node biopsy) procedure (*p* > 0.05).

**Conclusions:**

No changes in foot posture were observed in patients 5 years after the BCT procedure. The type of the surgical procedure related to the lymph nodes within the axillary fossa has no effect on changes in foot posture.

## Introduction

Breast cancer is the most common malignant tumor in female patients in the developed countries. The main therapeutic methodology consists of surgery encompassing mastectomy or breast-conserving therapy (BCT), depending on the indications. BCT consists in the excision of a focal lesion along with a margin of tissue not affected by cancer. Irradiation of the breast is an integral element of BCT [1, 2].

Results of randomized clinical studies reveal no differences in long-term treatment outcomes (e.g., overall survival) between patients subjected to BCT or mastectomy [3–5].

Treatment of patients with invasive forms of breast cancer requires verification of axillary lymph nodes. Depending on the baseline stage of the disease, sentinel lymph node biopsy (SLNB) or axillary lymph node dissection is required (ALND). Both procedures are associated with the risk of adverse effects [6, 7]. When the intervention within the axillary fossa is limited to the removal of the sentinel node, the incidence of adverse events is lower in a statistically significant manner. Long-term adverse effects of BCT-related surgery with axillary lymph node dissection include lymphedema of the upper limb, limited range of motion of the upper limb on the operated breast side, winged scapula, paresthesias, and postural defects [8, 9].

The physical and emotional activity of female patients after surgical treatment of cancer is reduced. This may lead to adverse changes within the motor system such as disturbed symmetry of shoulders and reduced range of motion of the upper limb within the shoulder joint (also due to irradiation of the region). These effects may also be due to reflexive pain-avoiding alignment of the limb. Disturbed range of motion within the shoulder joint is experienced by patients having undergone both BCT and mastectomy [10].

As demonstrated by Crosible et al., the asymmetry in shoulder position due to surgery leads to disturbed kinematics of the spine [11, 12]. Individual elements of human motor system are closely related to one another. Strains in one region of the body may be transferred onto other elements. Well-arched feet are an important element of correct body posture. Deformation of feet and the associated pain may result in postural instability and falls. As shown in our previous studies, differences in foot posture may be observed in patients undergoing mastectomy due to breast cancer between the operated and the contralateral side [13].

The objective of this study was to provide answers to the following questions:

- Are there any differences in the foot postures between the foot on the operated breast side and the foot on the contralateral side in patients having undergone BCT?
- Are there any differences in the load on the foot between the operated breast side and the contralateral side in patients having undergone BCT?
- Are there any differences in the foot postures and limb loads between the operated breast side and the contralateral side in patients having undergone BCT + ALND vs. patients having undergone BCT + SLNB?

## Materials and methods

The case–control study was carried out from April through June of 2017 following an approval no. 339/2017 being obtained from the Bioethics Committee of the Nicolaus Copernicus Medical College. Patients included in the study group had undergone surgical treatment for breast cancer at the Oncology Centre in Bydgoszcz in the period between 01.01.2011 and 30.12. 2011 (i.e., patients in whom 5 years had passed from the date of the surgical procedure). A total of 387 breast-conserving surgeries were carried out at our site in the aforementioned period. Following the analysis of medical documentation and qualification of patients on the basis of the inclusion and exclusion criteria as listed above, a total of 230 patients were qualified for the study. The chosen patients were invited by phone to participate in an additional free examination to assess the architecture of feet. Positive response was obtained from 166 patients. After the interview and physical examination (assessment of lymphedema, history of trauma or surgical procedures that might have affected the foot posture, postural defects, intake of balance-affecting drugs), a total of 116 patients were included in the analysis.

Study inclusion criteria—the following patients were included:

- having undergone BCT surgery with sentinel lymph node biopsy (SLNB) or axillary lymph node dissection (ALND) at the Oncology Centre in Bydgoszcz in 2011;
- in the age range of 40–70 years;
- with primary breast cancer;
- who provided informed consent for participation in the study;
- with appropriate physical fitness and no difficulties in walking.

Study exclusion criteria—the following patients were excluded:

- who required the scope of the surgical treatment being extended during the 5 years after the initial surgery to include axillary lymphadenectomy or mastectomy;
- with history of postural defects and trauma that might have affected foot posture;
- with the presence of other cancers;
- with lymphedema detected on examination;
- with mental disorders;
- with intake of drugs affecting the body balance;
- with other severe disorders (ASA class IV).

The study consisted of the sequence of the following elements:

- analysis of medical documentation (clinical staging, histopathological report);
- collection of data on supportive treatment;
- measurement of body mass and height with BMI calculation;
- measurement of the circumference of upper limbs on the operated breast side and on the contralateral side (using a tape measure at three upper limb levels: I—10 cm above the lateral epicondyle of the radial bone, II—10 cm below the lateral epicondyle of the radial bone, III—within the medial part of the metacarpus (excluding the tongue). Edema was defined as at least 2 cm difference between the circumference of the limb on the operated breast side and the contralateral limb at least one point of measurement;
- dual scales test to determine the load (in kilograms) on the left and the right foot (the test was carried out using two medical scales with the patient simultaneously placing both feet on both scales);
- assessment of the foot posture using a computer-based foot analysis tool as an extension of projection moiré (CQ Electronic)-based podoscopic examination; the assessment method was harmless and non-invasive for patients, who placed their bare feet on the device adopting a relaxed posture; the study lasted about 1 min with the examiner receiving the image of the patient’s feet displayed on the computer screen for further analysis of foot posture parameters; the parameters were transferred into an Excel worksheet.

The following foot posture parameters were determined using the chosen equipment:

- foot length (L) in mm,
- foot width (W) in mm,
- the Wejsflog index, L/W, to assess the transverse arch of the foot. The Wejsflog index ranges were defined as high arch (> 3.0), normal arch (2.44-3.0), and flat foot (< 2.44);
- ALPHA angle (hallux valgus angle)—normal range of 0–9º; values above 9º are indicative of hallux valgus;
- BETA angle (little toe varus angle)—normal range of 0–5º; values above 5º are indicative of varus toe;
- heel angle (GAMMA)—measured in degrees (º) to assess the transverse arch of the foot: high arch (< 15º), normal arch (15–18º), flat foot (> 18º);
- Sztriter–Godunov index (KY)—measured in degrees (º) to assess the longitudinal arch of the foot: hollow foot (0.00-0.25º), normal foot (0.26–0.45º), fallen foot (0.46–0.75º);
- Clarke’s angle—measured in degrees (º) to assess the longitudinal arch of the foot: hollow foot (> 55º), normal foot (42º–54º), fallen foot (20º–41º), and flat foot (< 20º);
- heel width (HW) in mm;
- area of foot adhering to the ground surface (FA)—in mm^2^.

Selected parameters were obtained from the image of the left and the right foot displayed on the computer screen. The results were listed and compared.

### Statistical analysis

Statistical analyses were carried out using PQStat statistical package, version 1.6.4.110.

Body mass and BMI before and after surgery were compared using the Student’s *t* test for dependent variables.

Comparisons of foot postures on the operated breast side and the contralateral side as a function of the type of intervention within the axillary fossa was carried out by means of variance analysis for repeated measures (with treatment as the grouping factor and the measurement side as the repeated measure) as well as Tukey’s post hoc test.

Differences, distributions, and results in scales categorized according to normative values depending on the measurement side were compared by the Chi square independence test.

Significance was defined as corresponding to the test probability of *p* < 0.05, while high significance was defined as corresponding to the test probability of *p* < 0.01.

## Results

The mean age of patients qualified to the study was 58.75 years. Out of the total population of 116 patients, 56 surgeries were performed on the left breast, while the remaining 60 surgeries were performed on the right breast of the patient. Sixty patients had undergone breast-conserving therapy with sentinel lymph node biopsy (BCT + SLNB group), while 56 patients had undergone breast-conserving therapy with axillary lymph node dissection (BCT + ALND group).

All patients declared a history of participation in motor rehabilitation classes; 46 patients were rural residents as compared to 70 patients residing in urban areas. Body mass and BMI values measured before surgery (data collected from medical documentation) and 5 years after surgery were compared in the study group. Body mass and BMI values increased significantly 5 years after surgery (*p* < 0.0002). Table 1 presents the detailed clinical characteristics of the study group. All patients testing positive for the presence of estrogen receptors (ER +) were qualified to hormone therapy (97 patients).

**Table 1:** Clinical characteristics of the study group

Variable	Results	
Age	*M* = 58.75, Me = 60.00, S.D. = 8.79	
Body weight before surgery	*M* = 70.42, Me = 69.00, S.D. = 11.91	*t* = − 3.9197
Body weight after surgery	*M* = 73.32, Me = 72.00, S.D. = 11.90	*df* = 115
		*p* = 0.0002
Height	*M* = 1.63, Me = 1.64, S.D. = 0.05	
BMI before surgery	*M* = 26.61, Me = 26.08, S.D. = 4.40	*t* = − 3.9180
BMI after surgery	*M* = 27.73, Me = 27.15, S.D. = 4.50	*df* = 115
		*p* = 0.0002
Operated side		
L	56 (48.28%)	
R	60 (51.72%)	
Procedure type		
BCT + SLNB	60 (51.72%)	
BCT + ALND	56 (48.28%)	
Menopausal status		
Yes	83 (71.55%)	
No	33 (28.45%)	
ER		
(+)	97 (83.62%)	
(−)	19 (16.38%)	
PR		
(+)	85 (73.27%)	
(−)	31 (26.72%)	
HER2		
(−)	49 (42.24%)	
(+ 1)	50 (43.10%)	
(+ 2)	2 (1.72%)	
(+ 3)	15 (12.93%)	
Clinical stage		
I A	86 (74.14%)	
II A	22 (18.97%)	
II B	8 (6.90%)	
Number of dissected nodes	*M* = 7.56, Me = 3.00, S.D. = 8.43	
Number of affected nodes	*M* = 1.19, Me = 0.00, S.D. = 3.23	
Supplementary treatment		
RTH	62 (53.45%)	
CHTH + RTH	54 (46.55%)	

*M* arithmetic mean, *S.D* standard deviation, *Me* median, *BMI* body mass index, *L* left, *R* right, *BCT* *+* *SLNB* breast-conserving therapy + sentinel lymph node biopsy, *BCT* *+* *ALND* breast-conserving therapy + axillary lymph node dissection, *ER* estrogen receptor, *PR* progesterone receptor, *HER2* human epidermal growth factor receptor 2, *RTH* radiotherapy, *CHTH* chemotherapy

Foot posture was assessed in the study group on the operated breast side—F1, as well as on the contralateral side—F2. Statistical analysis of this group of variables revealed no statistically significant differences for the following parameters: W, L/W, ALPHA, BETA, GAMMA, KY, Clark’s angle, HW, FA, WFA. Statistical differences were observed for limb load assessment (with higher load being placed on the foot of the healthy side; *p* = 0.0011), and L parameters (*p* = 0.0319, longer foot on the healthy side) (Table 2).

**Table 2:** Comparison of foot assessment parameters in the study patients

Tested parameter	F1			F2			Student’s *t* test	
	Mean	Median	Standard deviation	Mean	Median	Standard deviation	*t*	*p*
Foot load	36.00	34.75	6.54	37.23	36.50	6.01	− 3.3510	0.0011
L	224.85	227.00	12.83	225.66	227.00	12.67	− 2.1726	0.0319
W	86.59	86.00	6.25	87.01	88.00	6.02	− 0.9067	0.3664
Foot L/W	2.61	2.62	0.18	2.60	2.60	0.15	0.7638	0.4466
ALPFA	8.35	7.60	5.50	8.48	7.80	5.38	− 0.2760	0.7830
BETA	15.91	15.45	7.73	15.76	14.95	8.62	0.1762	0.8604
GAMMA	15.32	15,05	2.73	15.64	15.40	4.71	− 0.7354	0.4636
KY	0.27	0.20	0.24	0.29	0.30	0.25	− 0.5979	0.5511
CL	51.42	52.80	13.82	52.26	54.90	12.94	− 0.7726	0.4413
HW	51.23	51.45	4.83	51.46	51.05	5.65	− 0.6747	0.5012
FA	61.79	61.50	24.49	61.98	63.00	24.40	− 0.1750	0.8614

*F1* foot on the operated breast side, *F2* foot on the contralateral side, *L* foot length, *W* foot width, *L/W* Wejsflog index, *ALPHA* hallux valgus angle, *BETA* little toe varus angle, *GAMMA* heel angle, *KY* Sztriter–Godunov index, *CL* Clarke’s angle, *HW* heel width, *WFA* weighted foot area, *p* calculated probability value

No significant differences were observed for parameters characterizing the longitudinal and transverse arches of the feet either. In most women, the transverse arch was found to be within normal limits. The analysis of the longitudinal arch revealed hollow foot in most study patients (KY F1 = 51.72%; KY F2 = 49.14%)—Table 3.

**Table 3:** Comparison of categorized sizes of the transverse and the longitudinal arch of foot in the study patients

Transverse arch size	L/W				
	F1		F2		
	*N*	%	*N*	%	
High arch	2	1.724	1	0.862	Ch^2 = 2.6311
Normal arch	93	80.172	102	87.931	*df* = 2
Fallen arch	21	18.103	13	11.207	*p* = 0.2683

Longitudinal arch size	KY				
	F1		F2		
	*N*	%	*N*	%	
Hollow	60	51.72	57	49.14	Ch^2 = 0.8816
Normal	15	12.93	17	14.65	*df* = 3
Fallen	39	33.62	38	32.76	*p* = 0.8299
Flat	2	1.72	4	3.45	

*F1* foot on the operated breast side, *F2* foot on the contralateral side, *L/W* Wejsflog index, *KY* Sztriter–Godunov index, *p* calculated probability value

Categorized values of the ALPHA and BETA angles of both feet were analyzed in the study group. No statistically significant differences (*p* < 0.05) were observed for the results. ALPHA angles were normal in 60.34% of cases. Regardless of the side of the foot, hallux valgus was observed in similar percentages of patients (F1 39.65%, F2 37.93%). BETA angle assessment revealed varus little toes on the operated breast side in 93.97% and on the contralateral side in 90.52% of patients (Table 4).

**Table 4:** Comparison of categorized ALPHA and BETA angles in the study group

	ALPHA				*p*
	F1		F2		
	*N*	%	*N*	%	
0–9°	70	60.345	72	62.07	Ch^2 = 0.0726
> 9°	46	39.655	44	37.93	*df* = 1
					*p* = 0.7876

	BETA				*p*
	F1		F2		
	*N*	%	*N*	%	
0–5°	7	6.03	11	9.48	Ch^2 = 0.9637
> 5°	109	93.97	105	90.52	*df* = 1
					*p* = 0.3263

*F1* foot on the operated breast side, *F2* foot on the contralateral side, *ALPHA* hallux valgus angle, *BETA* little toe varus angle. *p* value of calculated probability

No statistically significant differences in the postures of both feet were observed in either the BCT + SLNB or the BCT + ALND group (*p* > 0.05) (except for the lower limb load in the BCT + ALND group patients who placed higher load on the foot on the side of the healthy breast (*p* = 0.042)). A similar lack of correlation was observed for the results obtained in both of these subgroups (BCT + SLNB vs. BCT + ALND)—Table 5.

**Table 5:** Comparison of foot postures in the study patients depending on the type of surgical procedure within the axillary fossa lymph node system

Tested parameter	BCT + SLNB, *n* = 60			BCT + ALND, *n* = 56			Comparison F1 vs F3, F2 vs F4, statistical significance
	Foot on operated side F1, mean	Foot on contralateral side F2, mean	*p*	Foot on operated side F3, mean	Foot on contralateral side F4, mean	*p*	
Lower limb load	36.38	37.43	0.1726	35.60	37.01	0.0422	*p*1 = 0.9080
							*p*2 = 0.9837
L	225.28	226.28	0.2247	224.39	225.00	0.6735	*p*1 = 0.9821
							*p*2 = 0.9491
W	86.75	87.33	0.7965	86.43	86.66	0.9850	*p*1 = 0.9923
							*p*2 = 0.9358
L/W	2.62	2.59	0.7334	2.61	2.61	0.9999	*p*1 = 0.9923
							*p*2 = 0.9763
ALPHA	8.62	8.88	0.9790	8.06	8.05	0.9999	*p*1 = 0.9438
							*p*2 = 0.8455
BETA	16.15	15.67	0.9790	15.66	15.85	0.9987	*p*1 = 0.9886
							*p*2 = 0.9994
GAMMA	15.24	16.07	0.5320	15.40	15.19	0.9866	*p*1 = 0.9962
							*p*2 = 0.6072
KY	0.28	0.27	0.9943	0.26	0.30	0.6764	*p*1 = 0.9887
							*p*2 = 0.9013
CL	52.54	51.49	0.8983	50.22	53.09	0.2584	*p*1 = 0.7895
							*p*2 = 0.9181
HW	51.16	51.47	0.9201	51.30	51.46	0.9899	*p*1 = 0.9988
							*p*2 = 1.0000
FA	59.41	60.08	0.9667	64.35	64.01	0.9958	*p*1 = 0.6961
							*p*2 = 0.8223

*BCT* *+* *SLNB* group undergoing BCT + SLNB surgery, *BCT* *+* *ALND* group undergoing BCT + ALND surgery, *F1* foot on the operated breast side, *F2* foot on the contralateral side, *L* foot length, *W* foot width, *L/W* Wejsflog index, *ALPHA* hallux valgus angle, *BETA* little toe varus angle, *GAMMA* heel angle, *KY* Sztriter–Godunov index, *CL* Clarke’s angle, *HW* heel width, *WFA* weighted foot area, *p1* F1 vs F3, *p2* F2 vs F4, *p* calculated probability value

## Discussion

The study assessed the changes in the load and posture of feet in women having undergone BCT 5 years after the procedure. The analysis revealed that 5 years after BCT, female patients placed higher loads on the side of the healthy breast (statistical significance of *p* = 0.0011 for comparison with the operated breast side). The analysis revealed statistically significant differences in foot loads within the BCT + ALND, with higher loads being placed on the healthy feet (*p* = 0.0422). No statistically significant differences were observed with respect to the remaining foot posture parameters (W, L/W, ALPHA, BETA, GAMMA, KY, CL, HT) between the operated side and the contralateral side. As also demonstrated in the study, the type of surgical procedure within the lymph nodes of the axillary fossa (ALND vs. SLNB) did not lead to any permanent changes in the lower limb loads and postures of the feet on the operated breast side and the contralateral side.

No studies of the impact of BCT on changes in foot posture and lower limb loads could be found in the available bibliographic databases. The comparison of foot posture parameters was carried out at a distant time interval after the completion of surgical treatment—after 5 years of patient follow-up characterized by no recurrences of cancer.

Our study is a contribution to the discussion on the adverse consequences of breast-saving therapy in breast cancer patients. As demonstrated in previous analyses performed at our site, a risk of adverse events exists also in the case of breast-saving surgical procedures. The adverse effects include limited range of motion within the shoulder joint, lymphedema, and postural defects within the sagittal and frontal planes [8, 9, 14]. We were also able to demonstrate a correlation between the number of dissected nodes and the reduction in the range of motion within the shoulder joint as confirmed by other studies [15, 16]. However, systematic rehabilitation after surgical resection of breast cancer facilitates minimization of the adverse effects of the breast cancer treatment as regards limited range of motion within the shoulder joint or the presence of lymphedema [17, 18].

Proper foot posture is an important element ensuring overall postural stability. Foot posture deformations have an impact on reduced mobility and balance disorders. These factors have a direct effect on the increased risk of falls [19]. Foot posture changes with age [20, 21]. As shown by Canseco et al., foot posture is better in individuals who are in good physical shape and lead an active lifestyle [22]. As shown in previous studies, mastectomy was associated with changes in foot posture [13, 23].

Our studies revealed no changes in foot posture between the operated breast side and the contralateral side. However, a number of deviations from normal foot posture were observed in the study group. The analysis of the longitudinal arch revealed hollow foot on the operated breast side in 51.72% and on the contralateral side in 49.14% of study patients. The analysis of hallux valgus angle revealed hallux valgus in more than 1/3 of the study patients. Varus little toe was observed in 93.97% of the patients on the operated breast side and in 90.52% of the patients on the contralateral side.

Deformations within the skeletal system develop usually due to wearing inappropriate shoes or as a consequence of age-related changes [24]. Foot posture in women depends on a number of factors such as body mass. Wearing high-heeled shoes has a negative effect on the architecture of feet [25, 26]. Our studies demonstrate the need to introduce exercises to improve proper foot arches similar to these in the post-mastectomy rehabilitation program. Rehabilitation activities should include education on proper shoe wearing and foot care.

Yang et al. point to the necessity of diagnosing, monitoring, and treating potential adverse effects regardless of whether the patients had undergone complete axillary lymph node dissection (ALND) or only sentinel lymph node biopsy (SLNB) [10]. Our study to assess the differences in foot postures of patients treated for breast cancer evaluated the distant adverse effects of oncological treatment. The study compared the changes in foot posture in women having undergone surgery for breast cancer depending on the type of intervention within the axillary fossa (ALND vs. SLNB). No statistically significant differences were observed for the assessed parameters (*p* > 0.05).

As shown by the available data, women having undergone either BCT or mastectomy for their breast cancer reduce their everyday activity, which has a negative impact on the motor system and the perceived quality of life [27, 28]. An increase in the body mass is common in women treated for breast cancer, mainly as a consequence of reduced physical activity [29–31]. Supplementary treatment also contributes to mass increase [32]. In our study, we were also able to demonstrate a statistically significant increase in body mass and BMI values of patients compared to the values recorded before surgery (*p* < 0.0002). A total of 97 patients from the study group were qualified for adjuvant hormone therapy after surgical treatment.

In our study, we demonstrated a statistically significant difference between the load placed on the lower limb on the operated breast side and the load placed on the lower limb on the contralateral side (36.00 vs. 37.23). We also identified statistically significant differences within the BCT + ALND group, with more load being placed by the patient on the lower limb on the operated side (*p* = 0.042).

The differences in the loads placed on lower limbs might be due to the changes in body posture, muscular imbalance, or the lower limb being positioned to avoid pain, particularly as relevant observations were more pronounced in the BCT + ALND group. In previous studies, the difference in the loads placed on the lower limbs was higher in the case of patients after mastectomy 13.

In our study, we also observed a statistically significant difference in the lengths of the feet on the operated side as compared to the contralateral side (224.85 mm vs 225.66 mm, *p* = 0.0319). This difference may reflect an ontogenetic trait not related to the surgical procedure.

The presented study has certain limitations, mainly consisting in a quite small population of patients included in the analysis. No evaluation of the architecture of feet before surgery had been performed either. Therefore, to obtain a full perspective on the changes in foot posture in breast cancer patients undergoing breast cancer treatment, a prospective study with an appropriately long clinical observation period should be conducted.

Our study showed that breast conservation treatment causes no adverse changes in foot posture on either the operated breast side or the contralateral side. No adverse effects of the treatment were also observed after a distant time interval following its completion (5 years after the surgical procedure). Thus, the well-known saying that “less is better” has been confirmed once again.
